# Effect of cooking methods on the nutritional quality of selected vegetables at Sylhet City

**DOI:** 10.1016/j.heliyon.2023.e21709

**Published:** 2023-10-28

**Authors:** Abdur Razzak, Tasnima Mahjabin, Md Rashedul Munim Khan, Murad Hossain, Ummay Sadia, Wahidu Zzaman

**Affiliations:** a Bangladesh Institute of Research and Training on Applied Nutrition (BIRTAN), Regional Office, Sunamganj, 3000, Bangladesh; b Department of Food Engineering and Tea Technology, Shahjalal University of Science and Technology, Sylhet, 3114, Bangladesh; c Department of Botany, University of Dhaka, 1200, Bangladesh

**Keywords:** Boiling, Steaming, Microwave cooking, Polyphenols, Heavy metal and minerals

## Abstract

The study was carried out to analyze the impacts of boiling, steaming, and microwave cooking on the physicochemical properties, the content of bioactive compounds, and boiling effect on mineral and heavy metal content of six widely consumed vegetables in Bangladesh's north-eastern region. In comparison to raw, boiled, and microwave-cooked vegetables, those that are steam-cooked retain a higher percentage of β-carotene with the exception of carrots. Boiling vegetables led to the most substantial reduction in ascorbic acid content (from 9.83 % to 70.88 %), with spinach experiencing the greatest decline. In contrast, microwaving had the mildest effect on ascorbic acid, preserving over 90 % of the initial content. The decrease in carotene content may be associated with color changes (decreasing greenness and increasing hue angle) in the chosen vegetables. The colorimeter shows the L* value (lightness/darkness) of all cooked vegetables significantly decreased. In terms of total polyphenol content (TPC) and total flavonoid content (TFC), boiling had a higher negative effect on most vegetables than the other two cooking methods, with losses of up to 70.3 % and 82.27 %, respectively. All cooked vegetables, with the exception of carrot and microwave pumpkin, had substantial reductions in free radicals scavenging activity, with losses ranging from 8.48 % to 56.73 %. In comparison to raw vegetables, boiled vegetables significantly lost minerals like potassium (K), magnesium (Mg), zinc (Zn), copper (Cu), and manganese (Mn). On the other hand, the calcium (Ca) and iron (Fe) content of all cooked vegetables, except for carrots and peas, exhibited an increase, ranging from 6 to 17 % and 6–12 %, respectively. The Cr concentration in all vegetables and the Zn, Fe, Mn, and Cd content in the spinach sample was higher than the FAO/WHO recommended maximum permissible level (MPL), whereas the accumulation of Cu and Ni content was lower in all vegetables. In conclusion, this study demonstrated that microwaving was the most effective method for retaining the nutritional value of vegetables, while steaming had a moderate impact.

## 1 Introduction

According to a household consumption survey, Bangladeshi people consume vegetable less than the recommended daily intake of 200 g, according to American Heart Association (AHA) which causes malnutrition more than half of the population has health difficulties [1]. Vegetable consumption is beneficial to human health since they are high in antioxidants, disease-fighting phytochemicals, sulfur-containing substances, minerals and vitamins [2]. Consumption of vegetables high in these functional components is linked to a lower incidence of chronic illnesses. The antioxidant capacity and phenolic compound concentration of fruits and vegetables appear to be linked to their health benefits. The ability of phenolic compounds to scavenge free radicals as well as reactive oxygen species has piqued curiosity, making them crucial in the prevention of oxidative processes that contribute to degenerative illnesses [3]. Vegetables have abundant amounts of vitamins such as β-carotene (pro-vitamin A), ascorbic acid (vitamin C), vitamins E and K etc. Among these, ascorbic acid has anti-aging and redox state regulatory effects as a potent antioxidant and β-carotene could be a synergistic antioxidant and may help to deter cancer [4,5]. Furthermore, the consumption of vegetables improves the immune system and alleviates conditions such as bronchitis, cataracts, asthma, and other respiratory syndromes [6].

Although vegetables are good source of antioxidants, vitamins and minerals such as chromium, nickel, selenium, zinc, they are susceptible to contamination of different chemicals for example heavy metals such as cadmium, mercury, lead, arsenic etc that accumulated in their leaves, roots, stem etc. In recent years, contamination of the food chain by heavy metals has become a major concern due to its significant accumulation in bio-systems via contaminated water, soil, and air [7]. Vegetable consumption has been identified as one of the key routes of human exposure to heavy metals in several studies [8,9]. Their presence in plant roots can disrupt plant quality and health by inhibiting or stimulating mineral uptake and metabolism in plant tissues [10].

There are a few vegetables that can be eaten raw, but most are cooked before they are consumed. The most prevalent household cooking methods include boiling, steaming, and microwave ovens [11]. In general, vegetables are cooked at home according to personal taste preferences and convenience, rather than with a focus on retaining their nutritional and health-promoting compounds. Recent studies have shown that the thermal treatments affect vegetables' physical, sensory, and bioactive aspects where both positive and negative outcomes are observed which depending on the type and quality of fresh vegetables and the cooking method chosen [12,13].

Only a few studies have been done on how cooking methods affect vitamin, mineral, heavy metal and phenolic component loss and retention [13,14]. This study aimed to determine the impact of various cooking techniques on bioactive compounds, physicochemical characteristics, mineral and heavy metal content as well as to estimate the percentage loss or retention of vitamins and antioxidant activities in selected vegetables widely consumed in Bangladesh's Sylhet region. In light of the potential toxicity and persistence of heavy metals, the frequency with which Bangladeshi inhabitants consume wild vegetables and the relevance of food safety, further research on wild vegetables of Bangladesh is still necessary. Therefore, the study also focused on the uptake of heavy metals in vegetables to provide recommendations regarding the consumption of vegetables cultivated in soils contaminated with heavy metals and cooking impact on the mineral and heavy metal content.

## 2 Material and methods

### 2.1 Sample collection

Two kg of fresh vegetable samples, consumed in Sylhet region including Carrot (*Dacus carota*), Sathkora (*Citrus macroptera*), Pumpkin (*Cucurbita maxima*), Green Peas (*Pisum sativum*), Pepper (*Capsium annum*), and Spinach (*Spinacia oleracea*), were procured from the Bangladesh Agricultural Development Corporation (BADC) Agro Service Center in Kumargaon, Sylhet, Bangladesh in 2022. To lessen bio variability, physically discolored, damaged, rotting samples as well as uneatable parts were discarded. The samples were then rinsed with tap water, then with distilled deionized water (DDW) to remove any adherent particles of dust, soil, single-celled algae and so on. Furthermore, the cleansed vegetables were dried with blotting paper. The samples were then sliced into 1 cm thin layers to attain the identical texture in the given cooking time and divided into 5 segments of 300g each, 4 of which were utilized to observe the cooking effect and 1 segment for mineral and heavy metal testing. This segment for mineral and heavy metal testing was oven-dried at (70 ± 1) ⁰C and then ground the dried vegetable using an electric grinder. The resultant powder was stored in desiccator in an airtight polythene pouch pending further analysis. All the samples were used in the cooking effect and analyzed within 15 h of preparation and extraction. The treatment time, amount of water and sample used are given in Table 1. After each treatment samples were drained of water and immediately chilled on ice. Sodium oxalate, hexane, methanol (96 %), Folin–Ciocalteu's phenol reagent, aluminium chloride (AlCl_3_), potassium acetate, 1,1-diphenyl-2-picrylhydrazyl (DPPH) were used in this study were of analytical grade and purchased from Sigma-Aldrich Chemical Co. (St. Louis, MO, USA).

**Table 1:** Treatment time (in min), amount of water (mL) and sample (g).

Treatment	Time(min)	Water(mL)	Amount of Sample(gm)
Raw	–	–	100
Boiled	6	150	100
Steamed	8	–	100
Microwave Cooking (900w)	1	6	100

### 2.2 Physicochemical analysis

#### 2.2.1 pH

Juice was extracted from the fresh, minced samples. The pH was then determined with a digital pH meter. (Model: HI-2211, Hanna Instrument, USA).

#### 2.2.2 Color measurement

Rana et al. (2020) [15] reported a method for determining the color of samples using a colorimeter (Model: PCE-CSM4, PCE instruments, UK). The apparatus was calibrated prior to the measurements. Fresh and treated were first washed and then chopped into small pieces. They are then blended until a smooth consistency is achieved. Then blended puree were placed on a glass plate to test their color values (H, L*, a*, b* and C*). The value of L* represents the lightness or darkness of a color where black is represented by L* = 0 and white by L* = 100. Similarly, the value of a* indicates the redness or greenness of a color where a value less than 0 represents greenness and greater than 0 represents redness. The value of b* reflects the yellowness or blueness of a color where a value greater than 0 indicates yellowness and less than 0 indicates blueness. All measurements were performed in triplicate the average results for each sample were tallied. The hue angle (H) and chroma (C) were determined by using equations (1), (2), (3) respectively.

(1) $$H=\tan^{-1}\left(\frac{b}{a}\right)\left(\text{when}\;a\;>\;0\right)\text{;}$$

(2) $$H={180+\tan}^{-1}\left(\frac{b}{a}\right)\left(\text{when}\;a\;<\;0\right)$$

(3) $$C=\sqrt{a^{2}+b^{2}}$$

### 2.3 Analysis of bioactive components

#### 2.3.1 Ascorbic acid (AA)

The total AA content of raw and cooked samples was determined using a modified version of the method described by Salkić et al. (2009) [16]. 1 g of material was homogenized (Model: HG-15A, Daihan Scientific Co. Ltd., Korea) with 10 mL of 0.056 M sodium oxalate for 2 min. The solution for extracting was left alone for 5 min. Then the homogenate solution was filtered and 0.5 mL of the extract's supernatant was combined with 0.056 M sodium oxalate to dilute it to 5 mL. Using 0.056 M sodium oxalate as a blank, the absorbance was measured with a UV–Vis spectrophotometer (Shimadzu, UV-1800, Japan) at 266 nm. Calibration curves were made with l-ascorbic acid as the standard.

#### 2.3.2 Determination of β-carotene

To measure overall carotenoids, 1 g of sample was homogenized in 10 mL distilled water for 2 min. Following that, 5 mL of hexane was added and rapidly stirred for 1 min, then left for 5 min to increase mass transfer, and finally stirred for 1 min. The absorbance of the resulting supernatant was measured at 452 nm using a UV–Vis spectrophotometer (Shimadzu, UV-1800, Japan) [17]. All investigations were done in triplicate, and the findings were presented as mg/100g using calibration curves of β-carotene as standard (y = 0.5442x + 0.0093; R^2^ = 0.9999).

#### 2.3.3 Extract preparation

To evaluate the bioactive compound, samples were extracted using the method presented by Zhang & Hamauzu (2004) [18], with some modifications. Samples (10.00 ± 0.02 g) were homogenized (Model: HG-15A, Daihan Scientific Co. Ltd., Korea) for 2 min in 40 mL of 60 % methanol. The samples were then incubated for 45 min at 100 RPM in a shaking incubator (SI-200, Korea). After that, the mixture was spun in a centrifuge (416G, Gyrozen, Korea) at 4000 rpm for 10 min and filtered through Whatman No. 4 filter paper. After standing in dark at 4 °C, the extract was used to measure TPC, TFC, and DPPH radical scavenging activities. Before analyzing a new batch, the stock solution was freshly prepared.

#### 2.3.4 Total polyphenol content (TPC)

According to Da Silva et al. (2011) [19], the total phenolic content (TPC) was determined using the Folin-ciocalteu phenol reagent. In brief, 0.5 mL extract was mixed with 8.5 mL distilled water and 0.5 mL Folin-ciocalteu phenol reagent. 1 mL of a 35 % sodium carbonate solution was added after the mixture had been left for 5 min. The mixture was then vortexed and stored for 20 min at room temperature. At 765 nm, the absorbance was measured using a UV–Vis spectrophotometer (Shimadzu, UV-1800, Japan). Gallic acid standard curve (y = 0.9028x + 0.054; R^2^ = 0.9998) was used to estimate the total phenolic content in the extract, which was expressed as Gallic acid equivalents (mg GAE/100g).

#### 2.3.5 Total flavonoid content (TFC)

To estimate the flavonoid content, 1.5 mL of 95 % ethanol, 0.1 mL of 10 % aluminium chloride (AlCl_3_), 0.1 mL of 1 M potassium acetate, and 2.8 mL of distilled water were mixed together and left the mixture in incubator at 25 °C for 40 min. After that, using deionized water as blank, the mixture absorbance was assessed in a UV–Vis spectrophotometer (Shimadzu, UV-1800, Japan) at 415 nm. The total flavonoid content was calculated from a calibration curve (y = 0.51x + 0.0268; R^2^ = 0.9941), and the result was expressed as quercetin equivalent (mg QE/100g) for the flavonoid contents(Chang et al., 2002) [20].

#### 2.3.6 Antioxidant activity (DPPH radical scavenging)

The antioxidant activity of the extract was assessed by the 2,2-diphenyl-2-picryl-hydrazyl (DPPH) radical scavenging assay [21]using UV–Vis spectrophotometer (Model-T60U, PG instruments limited, UK) at 517 nm. A solution containing no extract was employed as a blank reagent (A_control_). The following equation was used to determine the sample's ability to scavenge DPPH radicals.

$$\%\;\text{DPPH}\;\text{radical}\;\text{scavenging}\;\text{activity}=\left(\left(A_{\text{control}}–\;A_{\text{sample}}\right)/\;A_{\text{control}}\right)*\;100$$

where, A_control_ = the absorbance of blank; A_sample_ = the absorbance of the sample.

### 2.4 Determination of minerals and heavy metals

Atomic absorption spectrophotometer, flame emission spectrophotometer and UV spectrophotometer were used to identify and quantify minerals and heavy metals in the raw and boiled samples. Both raw and boiled vegetable samples were first washed with deionized water to remove any surface contaminants. The samples were then dried in an oven at 70 °C until a constant weight was achieved. The dried samples were then combusted in a muffle furnace at 550 °C for 6 h to produce ash. Each vegetable sample's ash residue was digested using a 1:4 solution of perchloric acid and nitric acid. After letting the samples cool, the contents were filtered through Whatman no 1 filter paper. Each sample solution was diluted with deionized water to a total volume of 25 mL. The mineral content of the aliquot was determined separately using an atomic absorption spectrophotometer (Spectra AA 220, USA Varian) [1].

### 2.5 Statistical analysis

Results have been analyzed using SPSS software (SPSS Inc., Chicago, IL, USA) and reported as the mean ± standard deviation (SD).

## 3 Results and discussion

### 3.1 The effect of cooking on physicochemical characteristics

Table 2 displays the pH of raw and cooked vegetables, with boiling spinach having the highest pH (6.65) and fresh sathkora having the lowest pH (1.79). In comparison to raw items, cooked sathkora (boiled, steamed, microwaved) and microwave cooked green pepper have a significant increase, whereas all other boiled vegetables have a slight increase. According to Monalisa et al. (2020) [22], the pH values of pumpkin, boiled at different times steadily increased may be due to the degradation of heat-sensitive and soluble acids during heating as well as reduction of active carboxylic groups in proteins, and the release of calcium and magnesium ions from proteins.

**Table 2:** Effect of cooking method on physicochemical properties.

Samples	Cooking treatments	p^H^	H	L*	a*	b*	c
Carrot	Raw	6.14 ± 0.06	63.45 ± 1.23	60.11 ± 1.25	22.55 ± 0.23	45.12 ± 1.23	50.44 ± 2.23
	Boiling	6.18 ± 0.04	55.71 ± 1.65	57.70 ± 1.83	24.20 ± 0.54	35.49 ± 1.76	42.96 ± 2.65
	Steam	6.06 ± 0.03	66.5 ± 1.76	56.80 ± 0.76	18.37 ± 0.42	42.24 ± 2.63	46 ± 1.56
	Micro wave	5.92 ± 0.02	63.54 ± 0.87	60.03 ± 0.63	20.00 ± 1.02	40.20 ± 1.93	44.90 ± 2.23
Pumpkin	Raw	6.31 ± 0.05	78.34 ± 0.65	55.11 ± 0.93	10.73 ± 0.54	51.98 ± 1,23	53.07 ± 2.65
	Boiling	6.50 ± 0.07	76.69 ± 1.07	50.35 ± 1.78	8.34 ± 0.87	35.28 ± 1.24	36.25 ± 1.67
	Steam	6.43 ± 0.09	82.49 ± 2.9	53.91 ± 2.24	4.59 ± 0.59	34.86 ± 2.34	35.1 ± 2.39
	Micro wave	6.11 ± 0.08	83.14 ± 0.32	52.21 ± 0.32	6.13 ± 0.78	50.96 ± 1.94	51.33 ± 1.32
Pea	Raw	6.25 ± 0.04	103.92 ± 0.94	36.76 ± 0.94	−7.56 ± 0.24	30.49 ± 1.39	31.41 ± 1.94
	Boiling	6.52 ± 0.02	101.99 ± 1.39	35.11 ± 2.39	−7.91 ± 0.32	37.23 ± 1.29	38.06 ± 2.39
	Steam	6.48 ± 0.03	101.43 ± 0.89	31.51 ± 1.89	−6.59 ± 0.53	32.62 ± 1.28	33.28 ± 0.73
	Micro wave	6.44 ± 0.06	106.21 ± 1.06	30.01 ± 1.78	−9.55 ± 0.86	32.83 ± 1.12	34.19 ± 1.26
Spinach	Raw	6.43 ± 0.05	120.96 ± 1.23	35.68 ± 1.12	−4.51 ± 0.25	7.53 ± 2.23	8.78 ± 1.42
	Boiling	6.65 ± 0.02	109.42 ± 2.02	30.83 ± 2.73	−3.2 ± 0.22	9.008 ± 1.56	9.03 ± 1.02
	Steam	6.58 ± 0.03	96.71 ± 1.70	35.76 ± 1.26	−1.0 ± 0.73	8.47 ± 2.87	8.53 ± 1.23
	Micro wave	6.49 ± 0.01	121.39 ± 2.01	35.92 ± 2.87	−3.1 ± 0.71	5.08 ± 0.99	5.75 ± 1.11
Sathkora	Raw	1.79 ± 0.09	92.46 ± 2.09	51.47 ± 0.99	−0.43 ± 0.89	10.41 ± 2.73	10.42 ± 2.19
	Boiling	2.07 ± 0.08	90.63 ± 1.56	49.62 ± 2.37	−0.12 ± 1.23	10.63 ± 2.02	10.63 ± 1.56
	Steam	1.95 ± 0.02	93.31 ± 2.02	51.32 ± 2.02	−0.56 ± 0.92	9.61 ± 1.62	9.62 ± 2.42
	Micro wave	1.93 ± 0.03	97.2 ± 1.62	50.51 ± 1.62	−1.35 ± 0.53	10.54 ± 1.33	10.63 ± 1.32
Green pepper	Raw	5.18 ± 0.08	121.43 ± 1.23	60.44 ± 1.93	−11.57 ± 0.28	19.58 ± 1.62	23.03 ± 1.13
	Boiling	5.21 ± 0.06	92.35 ± 1.34	56.71 ± 2.34	−1.13 ± 1.06	28.65 ± 1.02	28.68 ± 2.34
	Steam	5.11 ± 0.01	105.55 ± 2.98	56.84 ± 3.02	−3.93 ± 1.01	15.96 ± 1.21	16.63 ± 1.98
	Micro wave	5.45 ± 0.09	124.93 ± 2.43	58.63 ± 1.76	−11.45 ± 0.49	15.24 ± 1.76	19.12 ± 2.23

-Values in the table presented as mean ± Standard deviation of triplet evaluation.

The colorimetric values of raw and cooked samples summarized in Table 2. Consumer food preference and purchasing behaviour are influenced by color, which is one of the highest quality aspects of vegetables. To improve the quality and characteristics of food, color analysis and evaluation are required after harvesting and processing. Many pigmented components can be found in fruits and vegetables, including chlorophyll giving them a green color and carotenoid providing them an orange or yellow color [23]. The hue angle value varies depending on the type of vegetables and the cooking method. In Tables 2 and it can be shown that the hue angle of boiled and steamed spinach and green peppers declined considerably in comparison to raw. On the contrary, a significant rise in hue angle is found in steam and microwave cooked pumpkin and microwave cooked sathkora. The remaining microwave cooked vegetables had a modest rise in hue angle, indicating a rise in brightness. In comparison to raw and other cooking methods, the hue angle of microwave cooked greeny vegetables changed toward greener. Changes may be linked to the formation of chlorophyll derivatives, which do not alter the characteristics of chromophores or their progenitors' color [24]. It could also be owing to the microwave's short exposure time. Turkmen et al. (2006) [24] noticed a similar hue angle change after boiling (5 min), steaming (7.5 min), and microwave cooking (1.5 min) for various greeny vegetables. Significant reduction in redness (a*) and yellowness (b*) as well as an increase in hue angle (H) observed in cooked carrot and pumpkin. As previously reported [25] for cooked carrots, this decline in both redness and yellowness indicates a color loss mostly due to a decrease in β-carotene and their isomerization. Thus, these colour changes (a* decrease and H increase) could be related to the decrease of carotene content (Table 3). All cooked vegetables decreased in L* value which refers the darkening of the vegetable.

**Table 3:** Effect of cooking methods on β-Carotene and ascorbic acid (mg/100g FW).

Samples	Cooking treatments	β-Carotene (mg/100g FW)	Changes (%)	Ascorbic acid(mg/100 g FW)	Changes (%)
Carrot	Raw	8.02 ± 1.67	0.00	5.9 ± 1.23	0.00
	Boiling	7.41 ± 1.84	−7.64	5.32 ± 2.27	−9.83
	Steam	7.2 ± 1.76	−10.26	5.67 ± 1.82	−3.90
	Micro wave	7.56 ± 0.63	−5.77	5.88 ± 1.29	−0.34
Pumpkin	Raw	2.27 ± 0.93	0.00	30.91 ± 2.65	0.00
	Boiling	2.1 ± 0.78	−7.49	16.97 ± 1.67	−45.10
	Steam	2.42 ± 0.24	6.61 %	25.87 ± 2.39	−16.32
	Micro wave	2.11 ± 0.32	−7.05	29.45 ± 1.32	−4.73
Pea	Raw	3.08 ± 0.54	0.00	41.21 ± 3.94	0.00
	Boiling	2.86 ± 0.09	−7.14	27.98 ± 2.39	−32.10
	Steam	3.13 ± 0.06	1.63	35.64 ± 2.73	−13.52
	Micro wave	2.77 ± 1.78	−9.96	38.6 ± 4.26	−6.33
Spinach	Raw	4.9 ± 1.12	0.00	76.54 ± 2.42	0.00
	Boiling	3.32 ± 0.73	−32.22	22.29 ± 1.02	−70.88
	Steam	5.33 ± 0.26	8.71	30.33 ± 5.23	−60.37
	Micro wave	3.17 ± 0.87	−35.41	70.55 ± 3.11	−7.82
Sathkora	Raw	1.74 ± 0.99	0.00	60.26 ± 2.19	0.00
	Boiling	1.28 ± 0.37	−26.13	35.88 ± 3.56	−40.45
	Steam	1.87 ± 0.42	7.33	49.92 ± 2.42	−17.15
	Micro wave	1.68 ± 0.32	−3.58	59.33 ± 3.32	−1.54
Green pepper	Raw	1.82 ± 1.21	0.00	87.75 ± 2.13	0.00
	Boiling	1.12 ± 1.34	−38.46	65.13 ± 4.34	−25.77
	Steam	1.93 ± 1.02	6.04	49.43 ± 3.98	−43.66
	Micro wave	1.09 ± 0.76	−40.11	84.23 ± 4.23	−4.02

-Values in the table presented as mean ± Standard deviation of triplet evaluation. FW: Fresh Weight.

### 3.2 The impact of cooking methods on bioactive compound

#### 3.2.1 β-Carotene

Carotenoids are precursors of vitamin A and comprise β-carotene, lycopene and cryptoxanthin that are transformed to vitamin A in the body. β-carotene responsible for more than 90 % of overall carotenoids in plants and is essential for a significant amount of vitamin A activity. Table 3 displays the β-carotene content of raw and cooked vegetables, where the range for raw vegetables is 1.18–8.02 mg/100g fresh weight. It is apparent that the carotene content of all vegetables cooked with boiling and microwave reduced dramatically. The current study is in line with the findings of (Lee et al., 2018; Monalisa et al., 2020), where a comparable reduction had been discovered in cooked vegetables compared to fresh vegetables. This decrease could be explained by the varied intracellular locations of β-carotene. Vegetables were found to contain β-carotene in crystalline chromoplasts with polar lipid-rich membranes. Carotenes' physical state may change as a result of thermal processing of vegetables. In specifically, boiling allowed cellular lipids to solubilize carotenes [26]. Additionally, the amount of carotene lost due to dripping during the cooking process may be the source of the variation in the β-carotene retention of cooked vegetables. However, except carrot, vegetables cooked with steam have a considerable rise in β-carotene, which is consistent with recent research [13,26], where steaming spinach, green bean, broccoli, and mustered leaf have a much higher β-carotene content than raw product. This changes may be attributed to heating which may cause isomerization of the native trans form to its cis isomers, which are easily soluble in micelles, increasing the bioavailability and bio accessibility of carotene [22].

#### 3.2.2 Ascorbic acid (AA)

Ascorbic acid (AA), often known as vitamin C, is an essential water-soluble vitamin. It is obvious from this study's results that AA concentration varies greatly across the raw vegetables tested (5.90–87.75 mg/100g fresh weight), which are shown in Table 3. In this study, boiling treatment occurred in the most significant reduction of all tested vegetables, with losses ranging from 9.83 % to 70.88 %, with spinach exhibiting the highest reduction. After boiling, vegetables cooked with steam undergo a significant loss ranging from 13.52 % to 60.37 %. Rapid oxidation leads to dehydroascorbic acid conversion, followed by hydrolysis to 2, 3-diketogulonic acid and ultimately polymerization, which reduces AA during the thermal process. Plant tissues are leaching into the boiling water, contributing to the reduction [27]. Contrarily, microwaving vegetables had the least impact on AA with greater retention (>90 %). Due to the minimal water contact during low-temperature cooking, vegetables cooked in a microwave retain more of their vitamins than those that are boiled. According to Yang et al. (2019) [28], boiling broccoli for 5 min resulted in a greater loss of vitamin C than steaming and microwaving.

#### 3.2.3 Total phenolic compound (TPC)

The antioxidant properties of plant extracts come mostly from phenolic compounds, and the beneficial effects of phenolic compounds have been linked to their antioxidant capacity [29]. So, as far as the cooking effect on the TPC of vegetables is concerned, various cooking practices indicated unusual effects (Table 4). The TPC of several vegetables, such as pumpkin, pea, spinach, shatkora, and green pepper, is more significantly decreased by boiling than by the other two cooking techniques, with a loss of up to 70.3 %. It has already been observed that boiling decreases phenol content for a number of vegetables [30]. All steamed and microwaved vegetables, with the exception of carrots and pumpkins, have a deleterious effect on phenolic compounds. This decline could be attributed to the decomposition of heat-sensitive phenolic compounds and the leaching of water-soluble antioxidants into the cooking water [31]. The emergence of free phenolics from the hydrolysis process of tannins, driven by the greater pressure and higher temperature during steam cooking and/or microwave irradiation, may account for the considerable increase in total phenolic content (TPC) of cooked carrots and microwave-cooked pumpkins [32]. M. R. Rana et al. (2021) [13] also found an increase in phenolic compounds in green bean, cabbage, and mustard leaf under different cooking processes.

**Table 4:** Effect of cooking methods on TPC, TFC (mg/100g FW) and RSA (%).

Samples	Cooking treatment	TPC (mg/100g)	Changes (%)	TFC (mg/100g)	Changes (%)	DPPH RSA (%)	Changes (%)
Carrot	Raw	52.28 ± 1.54	0.00	5.39 ± 2.23	0.00	32.86 ± 1.23	0.00
	Boiling	55.82 ± 2.26	6.78	5.16 ± 1.27	−4.27	38.62 ± 3.37	17.52
	Steam	55.72 ± 1.12	6.6	4.78 ± 3.82	−11.32	43.00 ± 2.42	30.84
	Micro wave	77.97 ± 3.29	49.15	4.19 ± 2.29	−22.26	41.43 ± 2.29	26.07
Pumpkin	Raw	30.2 ± 2.95	0.00	19.67 ± 2.65	0.00	28.33 ± 1.65	0.00
	Boiling	16.23 ± 1.67	−46.26	15.87 ± 3.67	−19.32	20.42 ± 1.67	28.67
	Steam	17.32 ± 2.29	−42.6	18.26 ± 1.39	−7.17	25.88 ± 2.39	63.28
	Micro wave	48.27 ± 1.32	59.83	17.24 ± 3.32	−12.35	33.21 ± 1.32	17.23
Pea	Raw	158.17 ± 2.14	0.00	4.46 ± 2.94	0.00	25.84 ± 3.94	0.00
	Boiling	46.96 ± 2.39	−70.31	3.26 ± 1.39	−26.91	18.82 ± 2.29	−27.17
	Steam	82.41 ± 1.83	−47.90	4.32 ± 3.73	−3.14	26.41 ± 2.12	2.17
	Micro wave	87.28 ± 3.62	−44.82	1.16 ± 2.26	−73.99	15.73 ± 4.21	−39.13
Spinach	Raw	137.79 ± 1.23	0.00	53.36 ± 2.42	0.00	61.27 ± 2.32	0.00
	Boiling	64.24 ± 1.92	−53.38	9.46 ± 4.02	−33.21	39.54 ± 1.02	−35.47
	Steam	115.64 ± 5.23	−16.08	35.64 ± 1.23	−60.34	47.84 ± 2.13	−21.92
	Micro wave	99.68 ± 3.42	−27.65	33.66 ± 3.23	−36.92	51.23 ± 3.21	−16.39
Sathkora	Raw	291.53 ± 2.72	0.00	1.06 ± 1.19	0.00	63.23 ± 2.19	0.00
	Boiling	153.74 ± 3.94	−47.26	0.45 ± 0.56	−56.60	46.73 ± 3.56	−26.10
	Steam	180.32 ± 2.42	−38.15	0.68 ± 0.42	−35.85	50.12 ± 2.12	−20.73
	Micro wave	242.35 ± 3.32	−16.87	1.02 ± 0.32	−3.77	54.23 ± 3.32	−14.23
Green pepper	Raw	443.5 ± 2.42	0.00	32.23 ± 5.13	0.00	64.2 ± 4.13	0.00
	Boiling	345.14 ± 4.87	−22.18	27.8 ± 4.34	−13.74	39.46 ± 4.34	−38.53
	Steam	370.4 ± 3.21	−16.48	24.3 ± 3.98	−24.60	37.07 ± 1.98	−42.25
	Micro wave	287.99 ± 4.53	−35.06	17.21 ± 4.23	−46.60	27.78 ± 2.23	−56.73

-Values in the table presented as mean ± Standard deviation of triplet evaluation. TPC- Total phenolic content (mg/100g FW); TFC-Total flavonoid content (mg/100g FW); RSA-Radical Scavenging Activity (%).

#### 3.2.4 Total flavonoid content (TFC)

Fruits and vegetables receive a significant portion of their color and flavor from flavonoids, the most prevalent group of plant polyphenols. The total flavonoid content of raw and cooked vegetables is presented in Table 4, where it is apparent that raw spinach had the highest level (53.36 mg/100g) and that boiled shatkora had the lowest content (0.46 mg/100g). TFC, like TPC, has had a significant negative impact on boiled vegetables, with losses ranging from 4.27 % to 82.27 %. Although there has been a noticeable increase in TPC in cooked carrot, TFC has been observed to decline in all cooked carrot. This impact on TPC and TFC is in agreement with Bembem & Sadana, (2014) [14]. Steaming is the finest cooking method for preserving or extracting this phytochemical from vegetables, retaining it more effectively than boiling or microwaving. The previously mentioned explanation may be responsible for the negative effects of boiling and better retention in steaming [31,32].

#### 3.2.5 DPPH radical scavenging activity (RSA)

Measuring a substance's capacity to scavenge DPPH is a prominent way to estimate its antioxidant capacity. Antioxidants can change the color of DPPH from purple to yellow by accepting an electron or hydrogen radical [33]. Green pepper was the most active scavenger among the six vegetables tested, followed by shatkora, spinach, carrot, pea, and pumpkin (Table 3). Scavenging activity of vegetables varied according to cooking method and vegetable type. In comparison to raw vegetables, all cooked vegetables, with the exception of carrot and microwaved pumpkin, had a considerable negative influence on the scavenging activity, with losses ranging from 8.48 % to 56.73 %. Jaiswal et al. (2012) [34] observed similar lowering trends in various vegetables which could be attributed to the degradation and/or leaching of specific phenolic compounds or other chemicals responsible for DPPH RSA into boiling water during boiling.

According to research, phenolic substances, ascorbic acid, and carotenoids can all make a contribution to antioxidant activity [18]. In the current study, the overall antioxidant activity of the majority of vegetables decreased during cooking along with antioxidant components such ascorbic acid and total phenolic content. Because of the boiling process, cell walls and subcellular partitions are likely to be destroyed, allowing more components to be released [13]. However, cooked pumpkin and carrot have been found to have a significant rise, similar to TPC. These findings line up with a study that was done by (Miglio et al., 2008) [35]. The retention of radical-scavenging action in heated vegetables may also be related to the suppression of antioxidant oxidation by thermal inactivation of polyphenol oxidase and ascorbate oxidase [36].

#### 3.2.6 The effect of boiling on mineral and heavy metal content

The minerals K, Na, Ca, Mg, Fe, Zn, Cu and Cr of the raw and boiled vegetables are displayed in Table 5. The mineral content of vegetables exhibited varied tendencies, which may be ascribed to their existence in distinct forms in plant tissues, such as K as free form and Fe as bonded with protein or other high molecular compounds [37]. The mineral level of spinach was much higher than that of the other vegetables. However, the Zn, Fe, and Mn content of the spinach was over the maximum permissible level (MPL) recommended by the FAO/WHO organization(2001) [38], although the accumulation of Cu content was below MPL in all vegetables. It can be observed from the Table 5 that boiled vegetable had significant loss of minerals such as K, Mg, Zn, Cu, Mn in comparison with raw vegetables. The leaching effect that occurs during the cooking process may be to blame for the minerals in the vegetables being lost (Santos et al., 2018) [39]. On the other hand, all cooked vegetables, with the exception of carrot and pea, have an increase in Ca and Fe content that ranges from 6 to 17 % and 6–12 %, respectively. This increase could be attributed to binding to other food components, such as oxalates, which hinder extraction into the cooking liquid. Our results are in agreement with the results of García-Herrera et al. (2020) [37] who also found similar increment in Ca and Fe as well as decrement in K, Mg, Zn, Cu, Mn content.

**Table 5:** Effect of boiling method on Mineral content.

Sample	Treatment	K (mg/kg)	Ca (mg/kg)	Mg (mg/kg)	Zn (mg/kg)	Cu (mg/kg)	Fe (mg/kg)	Mn (mg/kg)
Carrot	Raw	32000	2600.43	1200	24	2.2	121	18.3
	Boiled	20000	2551.25	1122.5	19	2	98	16
Pumpkin	Raw	34000	1200.21	1200	17	3.2	24.2	12.5
	Boiled	20000	1356.25	943.75	8	3	27	9
Pea	Raw	24400	2500	4300	143	19	156	57
	Boiled	22100	2300	3900	119	17.6	147	41
Spinach	Raw	55800	5500.6	2300	121	14.2	850	967
	Boiled	27500	6162.5	1588.75	95	13	899	880
Sathkora	Raw	41000	1717.93	1400	14	2.7	168	7.2
	Boiled	25000	1813.75	1142.5	8	2	179	6
Green Pepper	Raw	42500	867	1800	37	10.2	145	78
	Boiled	36250	1015	1313.75	27	11	159	50
MPL					60	40	425.5	500

-MPL = Maximum permissible limit; K = potassium, Ca = calcium, Mg = Magnesium, Zn = zinc; Cu = cupper, Fe = iron, Mn = manganese.

The heavy metals concentrations of cadmium (Cd), lead (Pb), nickel (Ni), chromium (Cr)) obtained from the selected vegetables were listed in Table 6. The concentration of Cd and Ni were found to be highest in spinach which was recorded 4.48 and 12.2 mg/kg dry weight respectively. Sathkora was found the highest in chromium (Cr) which was recorded as 22.7 mg/kg whereas lowest in cadmium (Cd) content which was recorded as 0.08 mg/kg dry weight. However, there was no lead detected in any vegetable and no Cd detected in carrot as well.

**Table 6:** Effect of boiling method on heavy metal content of selected vegetable.

Sample	Treatment	Cd (mg/kg)	Loss (%)	Pd (mg/kg)	Loss (%)	Ni (mg/kg)	Loss (%)	Cr (mg/kg)	Loss (%)
Carrot	Raw	ND	0 %	ND	–	3.2	0 %	8.3	0 %
	Boiled	ND	0 %	ND	–	2.0	−38 %	7.7	−7%
Pumpkin	Raw	0.09	0 %	ND	–	4.9	0 %	6.7	0 %
	Boiled	0.06	−33 %	ND	–	2.5	−49 %	5.1	−24 %
Pea	Raw	0.12	0 %	ND	–	2.0	0 %	4.2	0 %
	Boiled	0.07	−42 %	ND	–	1.7	−14 %	3.9	−7%
Spinach	Raw	4.48	0 %	ND	–	12.2	0 %	18.3	0 %
	Boiled	3.62	−19 %	ND	–	10.0	−18 %	16.6	−10 %
Sathkora	Raw	0.08	0 %	ND	–	4.6	0 %	22.7	0 %
	Boiled	0.06	−25 %	ND	–	3.6	−21 %	20.7	−9%
Green Pepper	Raw	0.12	0 %	ND	–	7.1	0 %	7.6	0 %
	Boiled	0.05	−58 %	ND	–	5.3	−26 %	6.5	−15 %
MPL		0.2		0.3		67.9		2.3	

-MPL = Maximum permissible limit; ND- Not Detected; Cd = cadmium; Pd = lead; Ni = nickel; Cr = chromium.

It is clearly noticeable that all vegetables exceeded the maximum permissible limit of chromium. In addition, only spinach exceeded the maximum permissible limit (MPL) of Cd among the vegetables. On the other hand, the study result displayed that the level of accumulated Ni content in all tested vegetable species were below MPL value recommended by the FAO/WHO organization. Boiling exhibited reduction, ranging from 19 to 58 %, 14–49 %, 7–24 % in Cd, Ni and Cr respectively in selected vegetables (Table 6). The decreased quantities of such residues may be attributed to heavy metal solubilisation and volatilization during cooking process or the discharge of heavy metals outside the cooked tissues as free salts or in association with soluble amino acids and uncoagulated proteins [40]. The present findings were in agreement with those reported by Abd-Elghany et al. (2020) [40] who found a reduction tendency of heavy metal content in crabs and shrimps.

## 4 Conclusion

In general, boiling had the most detrimental impact on biochemical compounds, mineral and heavy metal contents. On the other hand, microwave cooking retained most of the AA, TPC, as well as steaming preserved most of the β-carotene. Additionally, compared to boiled and steamed vegetables, which impart more brightness, microwave-cooked vegetables demonstrated an upward tendency in hue angle (H). Cooked carrot and pumpkin showed a considerable reduction in redness (a*), while cooked pea and spinach showed an increase in greenness (-a*). All cooked vegetables experienced an obvious loss of lightness. After all, this investigation revealed that microwave cooking best preserved the nutritious value of vegetables, whereas steaming had a moderate effect.

## Data availability statement

Data included in article and no additional information is available for this paper.

## CRediT authorship contribution statement

**Abdur Razzak:** Writing – review & editing, Writing – original draft, Supervision, Methodology, Investigation, Funding acquisition, Formal analysis, Data curation, Conceptualization. **Tasnima Mahjabin:** Methodology. **Md Rashedul Munim Khan:** Writing – review & editing, Writing – original draft, Methodology, Formal analysis. **Murad Hossain:** Writing – original draft, Formal analysis. **Ummay Sadia:** Writing – original draft, Formal analysis. **Wahidu Zzaman:** Writing – review & editing, Writing – original draft, Supervision, Formal analysis, Conceptualization.

## Declaration of competing interest

The authors declare that they have no known competing financial interests or personal relationships that could have appeared to influence the work reported in this paper.
